# CT Angiography in the Lower Extremity Peripheral Artery Disease Feasibility of an Ultra-Low Volume Contrast Media Protocol

**DOI:** 10.1007/s00270-018-1979-z

**Published:** 2018-05-22

**Authors:** Barbora Horehledova, Casper Mihl, Gianluca Milanese, Rutger Brans, Nienke G. Eijsvoogel, Babs M. F. Hendriks, Joachim E. Wildberger, Marco Das

**Affiliations:** 1 Department of Radiology and Nuclear Medicine, Maastricht University Medical Center+, P. Debyelaan 25, PO Box 5800, 6202 AZ Maastricht, The Netherlands; 2 CARIM School for Cardiovascular Diseases, Maastricht University, Maastricht, The Netherlands; 3 Division of Radiology, Department of Medicine and Surgery, University of Parma, Parma, Italy; 4 Department of Diagnostic and Interventional Radiology, Helios Kliniken Duisburg GmbH, Duisburg, Germany

**Keywords:** Peripheral vascular diseases, Peripheral arterial disease, Diagnostic imaging, Multidetector computed tomography, Angiography, Radiographic image enhancement, Contrast media

## Abstract

**Purpose:**

The ALARA principle is not only relevant for effective dose (ED) reduction, but also applicable for contrast media (CM) management. Therefore, the aim was to evaluate the feasibility of an ultra-low CM protocol in the assessment of peripheral artery disease (PAD).

**Materials and methods:**

Fifty PAD patients were scanned on third-generation dual-source computed tomography, from diaphragm to the forefoot, as follows: tube voltage: 70 kV, reference effective tube current: 90 mAs, collimation: 192 × 2 × 0.6 mm, with individualized acquisition timing. The protocol ED (mSv) was quantified with dedicated software. CM protocol consisted of 15 ml test bolus and 30 ml main bolus (300 mgI/ml) injected at 5 ml/s, followed by a 40 ml saline chaser at the same flow rate. Aorto-popliteal bolus transit time was used to calculate the overall acquisition time and delay. Objective (hounsfield units—HU; contrast-to-noise ratio—CNR) and subjective image quality (four-point Likert score) were assessed at different anatomical regions from the aorta down to the forefoot.

**Results:**

Mean attenuation values were exceeding 250 HU from aorta down to the anterior tibial artery with CNR < 13. However, decline in attenuation was observed in more distal region with mean values of 165 and 199 HU, in left and right dorsalis pedis artery, respectively. Mode subjective image quality from the level of aorta down to the popliteal segment was excellent; below the knee mode score was good. The mean ED per protocol was 1.1 ± 0.5 mSv.

**Conclusion:**

Use of an ultra-low CM volume protocol at 70 kV is feasible in the evaluation of PAD, resulting in good to excellent image quality with mean ED of 1.1 ± 0.5 mSv.

**Level of evidence:**

Level 3, Local non-random sample

## Introduction

Peripheral arterial disease (PAD) is a slowly developing gradual narrowing of the vascular lumen caused by atherosclerosis, which is thereby limiting the blood flow in the affected area. Incidence of PAD is associated with age and an increased survival from coronary artery disease and stroke, which allows PAD to become symptomatic [1]. Patients with lower extremity PAD are clinically evaluated with help of an ankle brachial index [1, 2]. Computed tomographic angiography (CTA) can provide more detailed overview of the lower limb vasculature, rule-out or confirm an uncertain PAD diagnosis or verify the degree and an exact location of the stenosis prior to the revascularization [3–5]. The diagnostic value of CTA largely depends on the degree of intravascular contrast enhancement within the arterial segment of interest [6]. Moreover, imaging of the PAD has its additional specific technical challenges such as the extended scan range and the presence of vascular pathologies or abnormalities, which often lead to contrast timing issues. Previous studies showed that large CM volumes of up to 150 ml, accompanied by relatively high radiation doses, were used to overcome these difficulties [3, 4]. Naturally, this is not in accordance with the “As Low As Reasonably Achievable” (ALARA) safety principle, which is not only relevant for dose reduction, but also applicable for CM management.

The optimization of both scan and CM injection protocols in assessment of PAD is now possible as a result of a rapid technical advancement of computed tomography (CT) [7]. Lowering the CT tube voltage down to 70 kV results in a greater photoelectric effect of iodinated CM, as lower x-ray energies are getting closer to the k-edge of the iodine (33 keV). Compared to the standard 120 kV acquisition, the vascular enhancement of the same volume of iodinated CM increases by 25% at 100 kVp and by 70% at 80 kVp [8]. Moreover, patient’s size is not as dramatically pronounced in lower extremities, as it can be in abdominal area, and high tube current is not always necessary to keep the image quality diagnostic. Therefore, lowering the tube voltage settings allows for both a lower radiation dose and a CM volume reduction [9], possibly without compromising the image quality (e.g., with streak or blooming artifacts) [10–12], due to an increased vascular attenuation [13–15].

The aim of this study was to evaluate the feasibility of an ultra-low CM volume protocol in CTA assessment of the lower extremities vasculature, in combination with the bolus transit time technique and low-tube-voltage settings.

## Materials and Methods

### Study Population

Between November 2015 and October 2016, 50 patients referred for CTA of the peripheral arteries for clinical suspicion or evaluation of lower extremity artery stenosis, pre-interventional evaluation, post-operative follow-up were included in this study. General exclusion criteria for CTA examination applied (hemodynamic instability, pregnancy, renal insufficiency with glomerular filtration rate < 20 mL/min and iodine allergy). Patients with a history of lower extremity joint replacement received a dual energy (DE) scan protocol and were therefore also excluded from this study. Ethical approval and a waiver of informed consent were given by the local medical ethical research committee (ref.: 15-4-076). In accordance with the institutional review board guidelines data were coded and analyzed anonymously.

### CT Imaging Protocol

**Fig. 1:**
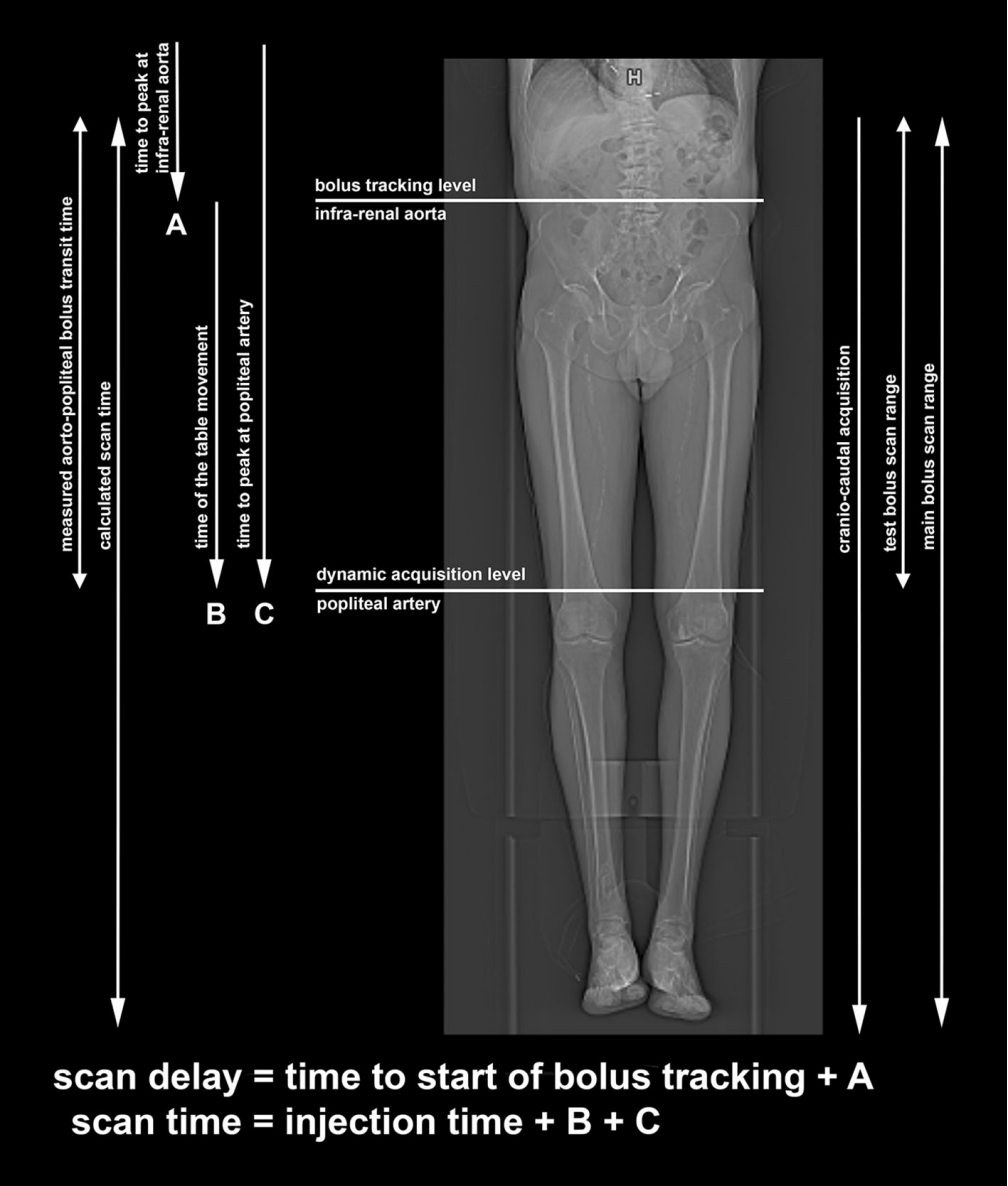
Scheme of imaging and contrast media protocol characteristics with scan delay and scan time calculation

All examinations were performed on a third-generation DSCT (Somatom FORCE, Siemens Healthcare, Forchheim, Germany). The imaging protocol is summarized in Table 1. The measured aorto-popliteal bolus transit time was used to calculate the scan time (injection time: 6 s + the time of the table movement: 5 s + the time to peak at popliteal artery (PA)) and scan delay (time to the start of bolus tracking: 8 s + the time to peak at the infra-renal aorta) from the diaphragm to forefoot (Fig. 1). The pitch value was adjusted according to the calculated scan time.

**Table 1:** Imaging protocol and contrast media application parameters

Scan type/direction	Helical/cranio-caudal
Scan range	Diaphragm to the forefoot
Tube voltage	70 kv
Reference quality tube current	90 mAs
Automated tube current dose modulation	CARE Dose4D
Rotation time	0.5 s
Pitch	Individual
Slice collimation	192 × 2 × 0.6
Slice width/increment	2/1.4 mm
Iterative reconstruction	Model-based algorithm (ADMIRE, Siemens Healthcare, Forchheim, Germany)
Reconstruction strength	Level 3
Reconstruction kernel	Bf40
Contrast media	Iopromide; 300mgI/ml (Ultravist; Bayer Healthcare, Berlin, Germany)—pre-warmed to 37 °C
Test bolus	15 ml CM * 5 ml/s 40 ml NaCl* 5 ml/s
Main bolus	30 ml CM * 5 ml/s 40 ml NaCl* 5 ml/s
Iodine delivery rate	1.5gI/s
Total iodine load	13.5gI (4.5 + 9)
Catheter size	18-22G

*CM* contrast media, *G* gauge, *gI* grams of iodine, *gI/s* grams of iodine per second, *kV* kilovolt, *mAs* milliampere-second, *ml* milliliter, *mm* millimeter, *NaCl* saline, *s* second)

### Injection Protocol

The CM protocol is summarized in Table 1. A double-level semi-manual test bolus technique was used to determine the aorto-popliteal bolus transit time, with first level at the infra-renal aorta and second level just above the knee. Pre-monitoring, with a circular region of interest (ROI) drawn at the infra-renal aorta, started 8 s after the test bolus was administered. When the threshold of 70 HU was detected at the ROI, the table automatically slid to the second level above the knee, where a dynamic acquisition of the same test bolus started immediately (Fig. 1). The dynamic acquisition was aborted manually when a decrease in intravascular attenuation following a peak was observed at the level of PA. Time-attenuation curves of both levels were assessed with dedicated software (Syngo DynEva™ Siemens Healthcare, Forchheim, Germany).

### Effective Dose Assessment

A dedicated dose monitoring software (Radimetrics™; Bayer Healthcare, Berlin, Germany) recorded the dose-related parameters (effective mAs, irradiated length, pitch, etc.) and calculated the ED in millisieverts (mSv) per scan, according to the Publication 103 of the International Commission on Radiological Protection (ICRP 103) [16].

### Peripheral Vascular Calcifications

The presence or absence of peripheral vascular calcifications was subjectively evaluated. Vascular calcifications were defined as linear, (semi-) circular or crescent hyperdensities of vascular wall. Cutaneous or other non-vascular soft tissue calcifications were not included. The extent of the calcifications was classified as follows: 1. Absent to little calcifications 2. Mild to moderate calcifications 3. Significant calcifications.

### Angiographic Evaluation

An interventional radiologist (RB) scored the vascular lesions at the aorto-iliac and femoro-popliteal segment according to the Trans-Atlantic Inter-Society Consensus Document II (TASC II, 2007) [17] into 5 groups (A-D lesion types and 0 for non-diseased segment).

### Objective CT Image Quality

The objective CT image quality was assessed, on transverse images with manually placed ROIs by a research fellow trained for this analysis (BH), at the supra- and infra-renal aorta and 5 levels of peripheral arteries in both, left and right limb, as follows: common iliac artery (CIA), superficial femoral artery (SFA), PA, ATA, DPA. The size of each ROI was drawn as large as possible approximately in the center of the length of the evaluated vessel segment and was carefully adapted to assess the entire vessel lumen without including the arterial wall, calcifications or plaques. Objective image quality was quantified as intravascular attenuation of the desired vessel, signal-to-noise ratio (SNR), and CNR. SNR was defined as vessel enhancement in HU divided by vessel enhancement standard deviation (SD). CNR was defined as vessel enhancement [HU] minus adjacent muscle tissue enhancement [HU], divided by adjacent muscle tissue enhancement SD [18]. Image quality at aorta was considered diagnostic for attenuation values > 200 HU [19, 20] and CNR > 3 [21]. The distal vessel segments were considered diagnostic, when the subjective image quality was graded as diagnostic or higher.

### Subjective CT Image Quality

An experienced radiologist (CM) and radiology resident (GM) evaluated the subjective image quality independently, blinded to each other, using a 4-point Likert scale; grade 1 = non-diagnostic (contrast enhancement not sufficient for diagnosis), grade 2 = diagnostic (image sufficient for diagnosis, although contrast enhancement is unsatisfactory), grade 3 = good (diagnostic image with satisfactory contrast enhancement), grade 4 = excellent (diagnostic image with optimal contrast enhancement). The minimum target vascular attenuation values in HU, needed to achieve a diagnostic enhancement (Likert score ≥ 2) at the peripheral vessels, were derived from the comparative analysis of the objective and the subjective image quality evaluation assessed in this study.

### Statistical Analysis

Statistical analysis was conducted using Statistical Package for Social Sciences version 23.0 (SPSS Inc., Chicago, IL, USA). Continuous variables are expressed as mean ± SD; categorical and ordinal variables are expressed as mode, frequencies or percentages. Normality of data distribution was evaluated using Shapiro–Wilk test. The independent *t* test was used to compare continuous and ordinal variables. The inter-observer agreement was quantified as a mean of a difference in assigned scores. One-way analysis of variance (ANOVA) was used to compare intravascular attenuation and CNR between TASC II groups in aorto-iliac and femoro-popliteal segments. The Bland–Altman methods with 95% confidence interval (CI; mean difference ± 1.96 × SD) were used to establish the difference in the intravascular attenuation between left and right vasculature. The linear regression analysis was then used to assess the proportional bias. Box plots visualize the intravascular attenuation among TASC II lesion types (GraphPad Prism version 5.04, GraphPad Software, San Diego, CA, USA). All *p* values are two-sided, and a *p* value below 0.05 was considered statistically significant.

## Results

### Baseline Characteristics and Effective Dose Assessment

Baseline characteristics are listed in Table 2 and the dose-related characteristics in Table 3.

**Table 2:** Baseline characteristics

Baseline characteristics	Mean ± SD	Range
Age (years)	68 ± 11	41–95
Weight (kg)	77 ± 17	45–103
Height (cm)	168 ± 10	148–186
BMI (kg/m^2^)	27 ± 5	17–37

Clinical presentation	*n* (%)	
Rutherford I	23 (46%)	
Rutherford II	12 (24%)	
Rutherford III	5 (10%)	
ALI	5 (10%)	
Other (aneurysma, peripheral embolization due to malignity/infection)	5 (10%)	

PAD risk factors	*n* (%)	
Smoking	36 (72%)	
Current smoker	21 (42%)	
Ex-smoker < 1 year	4 (8%)	
Ex-smoker < 1 year	11 (22%)	
Diabetes mellitus	16 (32%)	
Dyslipidemia	23 (46%)	
Hypertension	31 (62%)	
Family history of CVD	15 (30%)	

*ALI* acute limb ischemia,* BMI* body mass index,* cm* centimeter,* CVD* cardio-vascular disease,* kg* kilogram, *m* meter, *m*^2^ square meter,* PAD* peripheral artery disease

**Table 3:** Imaging and acquired radiation dose characteristics

Scan characteristics	Mean ± SD	Range
Mean tube current (mAs)	130 ± 57	56–295
Pitch	0.48 ± 0.12	0.35–0.7
Irradiated length (cm)	124.9 ± 12.4	104.2–149.7
Scan time (s)	29.3 ± 8.7	16–59
CTDI_vol_ (mGy)	3.20 ± 1.02	1.9–6.7
DLP (mGy*cm)	400.2 ± 142.2	209.6–876.2
ED (mSv)	1.1 ± 0.5	0.5–3.1
Bladder (mSv)	0.14 ± 0.04	0.1–0.32
Testes (mSv)	0.55 ± 0.21	0.3–1.14
Ovaries (mSv)	0.16 ± 0.05	0.07–0.27
Skin (mSv)	0.04 ± 0.02	0.02–0.1
Red bone marrow (mSv)	0.1 ± 0.04	0.04–0.25
Skeleton (mSv)	0.06 ± 0.02	0.03–0.14

*cm* centimeter,* kg* kilogram,* m* meter,* mAs* miliamperes,* mGy* miligray,* mSv* milisievert,* s* second

Study population consisted of 28 males (56%) and 22 females (44%), with the average age 68 ± 11 years. According to Rutherford classification, 23 patients (46%) had grade I, 12 (24%) had grade II, and 5 (10%) had grade III PAD. Five patients presented with acute occlusion, and 5 were referred with other clinical entities (aneurysm, pain during exercise, peripheral embolization due to malignity or infection).

The mean ED per protocol was 1.1 ± 0.5 mSv. Twenty-seven patients had significant peripheral vascular calcifications, 18 presented with mild to moderate and 5 patients with absent to little calcifications.

### Angiographic Evaluation

The distribution of TASC II lesion groups is summarized in Table 4. Bilateral, equally severe, pathologies were described in 17 patients (34%). In 18 patients, the vasculature of the left leg was more severely affected (36%), in 13 patients the right leg was more affected (26%). Endovascular intervention was planned based on the CTA in 24 patients (48%). Percutaneous transluminal angioplasty (PTA) was required in 12 (46%), stent implantation in 10 (38%) and intra-arterial thrombolytic therapy in four of these patients (15%). Twelve patients (24%) underwent surgery (9 had bypass, 2 had surgical thrombectomy, and one patient had an open surgical correction of AFC and AP aneurysm). One patient refused surgery. Supervised exercise therapy was prescribed in two patients (4%) and conservative approach was chosen in eight patients (16%). In one patient, no treatment option was available due to persistent wound infections, after extensive vascular reconstructions and amputations, and patient repeatedly requested euthanasia. Two patients had no vascular pathologies on CTA (one patient with post-procedural evaluation of femoro-popliteal bypass with good flow and one patient with normal but slim vasculature).

**Table 4:** Overview of distribution of the TASC II classification for the study population (50 right and 50 left legs) with respective intra-vascular attenuation (HU) and contrast-to-noise ratio (CNR) values

	Right leg								Left leg							
	Aorto-iliacal				Fem.-popliteal				Aorto-iliacal				Fem.-popliteal			
	*n*	%	HU	CNR	*n*	%	HU	CNR	*n*	%	HU	CNR	*n*	%	HU	CNR
0	33	66	366 ± 107	19 ± 8	20	40	386 ± 109	22 ± 11	32	64	384 ± 122	20 ± 12	21	42	363 ± 106	21 ± 12
A	7	14	452 ± 99	29 ± 12	6	12	339 ± 96	18 ± 8	7	14	404 ± 104	27 ± 12	5	10	347 ± 103	22 ± 16
B	2	4	585 ± 154	27 ± 9	11	22	344 ± 92	18 ± 14	4	8	432 ± 191	23 ± 8	8	16	396 ± 125	20 ± 10
C	4	8	374 ± 52	20 ± 6	10	20	372 ± 75	20 ± 8	3	6	375 ± 43	20 ± 7	9	18	373 ± 73	18 ± 6
D	4	8	370 ± 81	19 ± 7	3	6	333 ± 111	17 ± 8	4	8	402 ± 105	22 ± 7	7	14	349 ± 100	20 ± 12
Sign.			*p* = 0.001*	*p* = 0.006*			*p* = 0.401	*p* = 0.241*			*p* = 0.986*	*p* = 0.319*			*p* = 0.714	*p* = 0.990*

*logarithmic transformation to correct for non-normal distribution of data

### Objective CT Image Quality

**Fig. 2:**
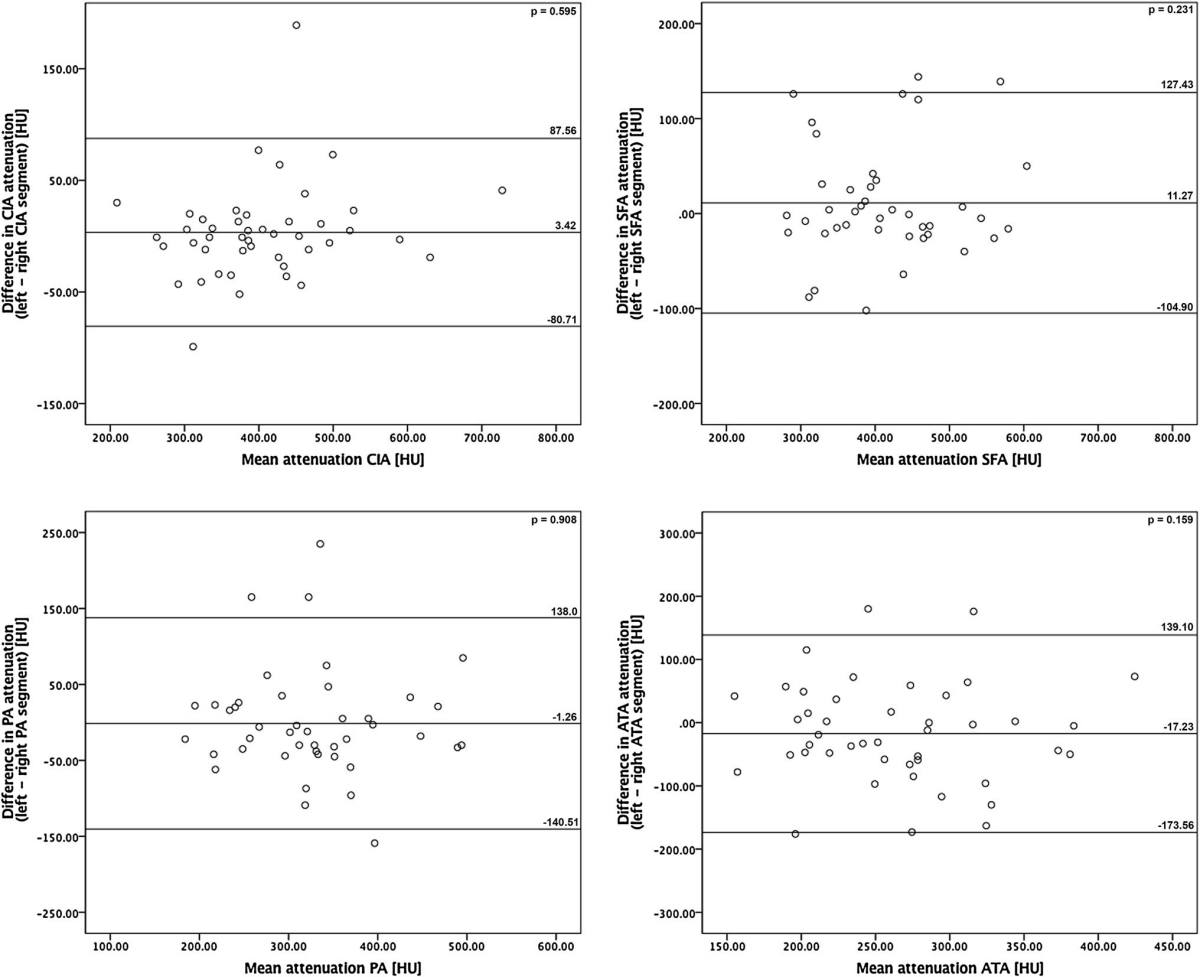
Bland–Altman plots demonstrating the difference in the intravascular attenuation between left and right vasculature at respective levels of the peripheral arteries, shown with 95 % confidence interval. The middle line presents the mean difference (expressed in HU), and the upper and lower lines represent 95 % confidence interval. *ATA* anterior tibial artery, *CIA* common iliac artery, *HU* hounsfield unit, *PA* popliteal artery, *SFA* superficial femoral artery, *p p* value)

**Fig. 3:**
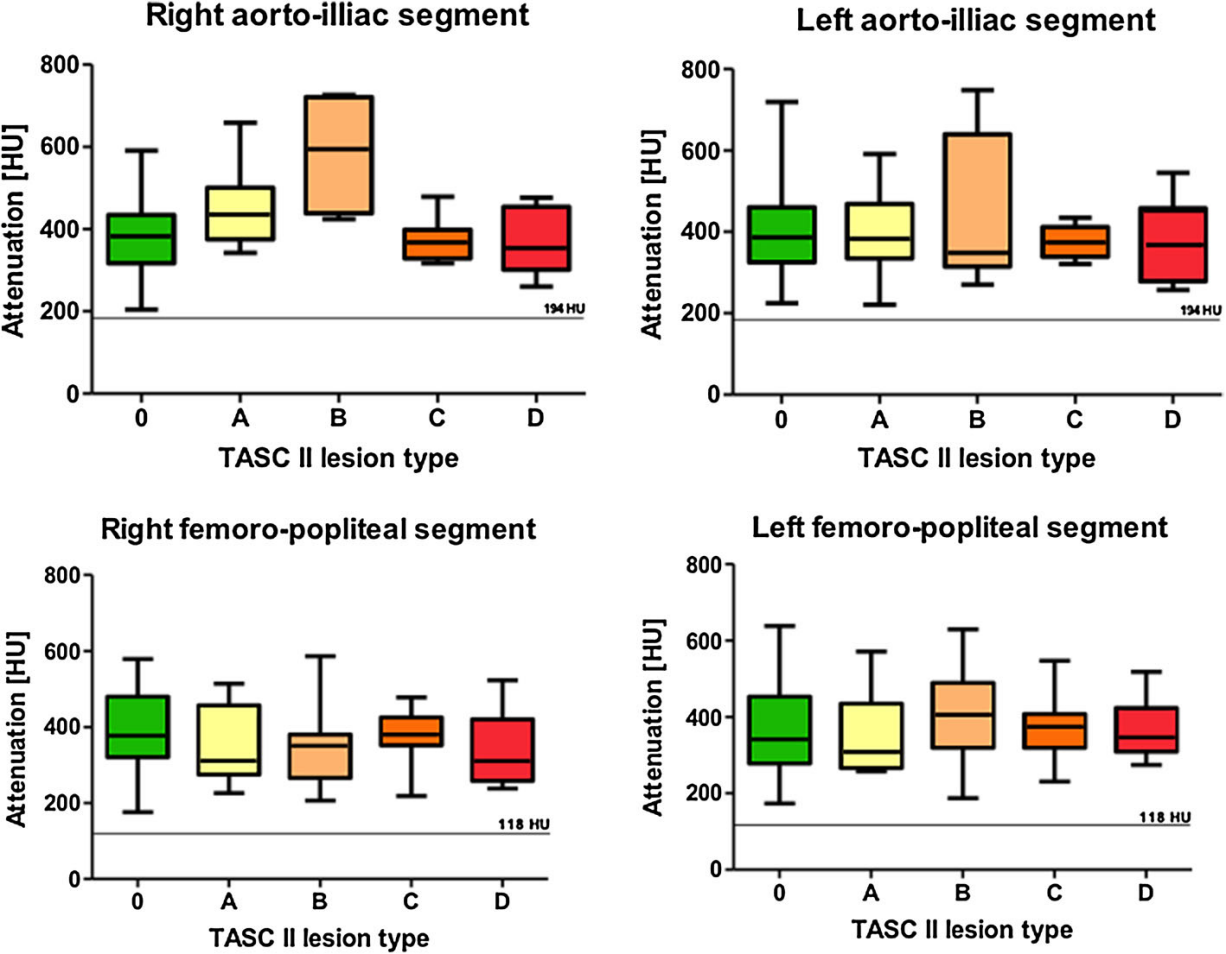
Box plots presenting the intravascular attenuation among TASC II lesion types in the aorto-iliac and femoro-popliteal segments. The threshold lines at the level of 194 HU (for aorto-iliac segment) and 118 HU (for femoro-popliteal segment) represent the minimum intravascular attenuation values recognized as diagnostic for the subjective image quality evaluation. *HU* hounsfield unit, *0* non-diseased segment, A–D TASC II—lesion types

Mean attenuation values from the infra-renal aorta down to the anterior tibial segment were all greater than 250 HU with a CNR greater than 13 (Table 5). In the ADP, the mean vascular attenuation was 199 ± 87 HU and 165 ± 65 HU, in right and left side, respectively, with a CNR greater than 7. In 49 out of 546 evaluated segments (9%), the attenuation could not be assessed due to a present vascular occlusion or due to a decreased attenuation in the segment distally from the occlusion. The number of complete unenhanced segments (vascular attenuation < 68 HU) on the left and right side were, respectively: CIA 3;2, SFA 5;3, PA 3;3, ATA 3;4, DPA 9;14. A statistically significant (*p* = 0.021) difference between the left and right intravascular attenuation was found only in the DPA segment (− 35 HU; left side more severely affected). The mean difference of 3 HU, 11 HU, − 1 HU and − 17 HU between left- and right-sided intravascular attenuation at the CIA, SFA, PA and ATA segment, respectively, was not statistically significant (*p* ≥ 0.159). There is no proportional bias (*p* ≥ 0.153) in the Bland–Altman plots of differences between left and right segments (Fig. 2). An overview of vascular attenuation at the aorto-iliac and femoro-popliteal segments for TASC II lesion groups is presented in Table 4 and Fig. 3. A statistically significant difference in the intravascular attenuation (*p* = 0.001) and CNR (p = 0.006) between TASC II groups was observed only in the right aorto-iliacal segment. In this segment, the mean intravascular attenuation (and CNR) in non-diseased vasculature and most severely affected group D were 366 ± 107 HU (19 ± 8) and 370 ± 81 HU (19 ± 7), respectively. No statistically significant differences were found in intravascular attenuation (and CNR) between TASC II severity types in left aorto-iliacal segment (*p* = 0.986) and both, left and right, femoro-popliteal segments (*p* = 0.714 and *p* = 0.401, respectively).

**Table 5:** The objective image quality summarized as mean vascular attenuation values, signal-to-noise ratio and contrast-to-noise ratio

	Attenuation ± SD (HU)	SNR	CNR	Significance (2-tailed)
Muscle	51 ± 17			*p* < 0.001
Supra- renal aorta	363 ± 100	17 ± 7	19 ± 8	*p* < 0.001
Infra- renal aorta	387 ± 103	21 ± 9	21 ± 9	*p* < 0.001
CIA				
L	414 ± 113	21 ± 10	23 ± 13	*p* < 0.001
R	405 ± 95	20 ± 8	22 ± 9	*p* < 0.001
SFA				
L	417 ± 91	20 ± 9	24 ± 13	*p* < 0.001
R	408 ± 91	20 ± 9	23 ± 12	*p* < 0.001
PA				
L	319 ± 90	19 ± 10	17 ± 8	*p* < 0.001
R	324 ± 87	20 ± 12	18 ± 9	*p* < 0.001
ATA				
L	252 ± 73	16 ± 10	13 ± 6	*p* < 0.001
R	270 ± 75	16 ± 11	14 ± 7	*p* < 0.001
DPA				
L	165 ± 65	12 ± 10	7 ± 5	*p* < 0.001
R	199 ± 87	15 ± 14	9 ± 5	*p* < 0.001

*ATA* anterior tibial artery, *CIA* common iliac artery, *CNR* contrast-to-noise ratio, *DPA* dorsalis pedis artery, *HU* hounsfield unit, *L* left side, *PA* popliteal artery, *R* right side, *SD* standard deviation, *SFA* superficial femoral artery, *SNR* signal-to-noise ratio)

### Subjective CT Image Quality

In total 546 segments were evaluated by two experienced readers (Table 6). The subjective image quality at the level of infra-renal aorta down to the popliteal segment was graded as excellent (mode Likert scale = 4). The subjective image quality below the knee was graded as good (mode Likert scale = 3). There was no significant difference between the subjective image quality scores graded by two readers (*p* ≥ 0.078). The minimum intravascular attenuation values recognized as diagnostic for the subjective evaluation (i.e., score greater than 2) were as follows: CIA ≥ 194 HU, SFA ≥ 227 HU, PA ≥ 118 HU, ATA ≥ 143 HU, DPA ≥ 94 HU.

**Table 6:** The subjective image quality summarized as mode of assigned Likert grades and mean difference between readers

Segment	Mode score per segment		Mode score Reader 1	Mode score Reader 2	Mode assigned score (2 readers)	Mean difference between readers	Sign.(2-tailed)
Aorta	4		4	4	–	0.08	*p* = 0.159
CIA	4	L	4	4	4	0.14	*p* = 0.110
		R	4	4	4	0.09	*p* = 0.103
SFA	4	L	4	4	4	0.11	*p* = 0.210
		R	4	4	4	0.19	*p* = 0.090
PA	4	L	4	4	4	0.14	*p* = 0.110
		R	4	4	4	0.13	*p* = 0.225
ATA	3	L	3	3	3	0.17	*p* = 0.164
		R	3	3	3	0.25	*p* = 0.078
D*p*A	3	L	3	3	3	0.13	*p* = 0.598
		R	3	2	2	0.4	*p* = 0.374

*ATA* anterior tibial artery, *CIA* common iliac artery, *DPA* dorsalis pedis artery, *L* left side, *PA* popliteal artery, *R* right side, *SFA* superficial femoral artery, *Sign*. significance

## Discussion

**Fig. 4:**
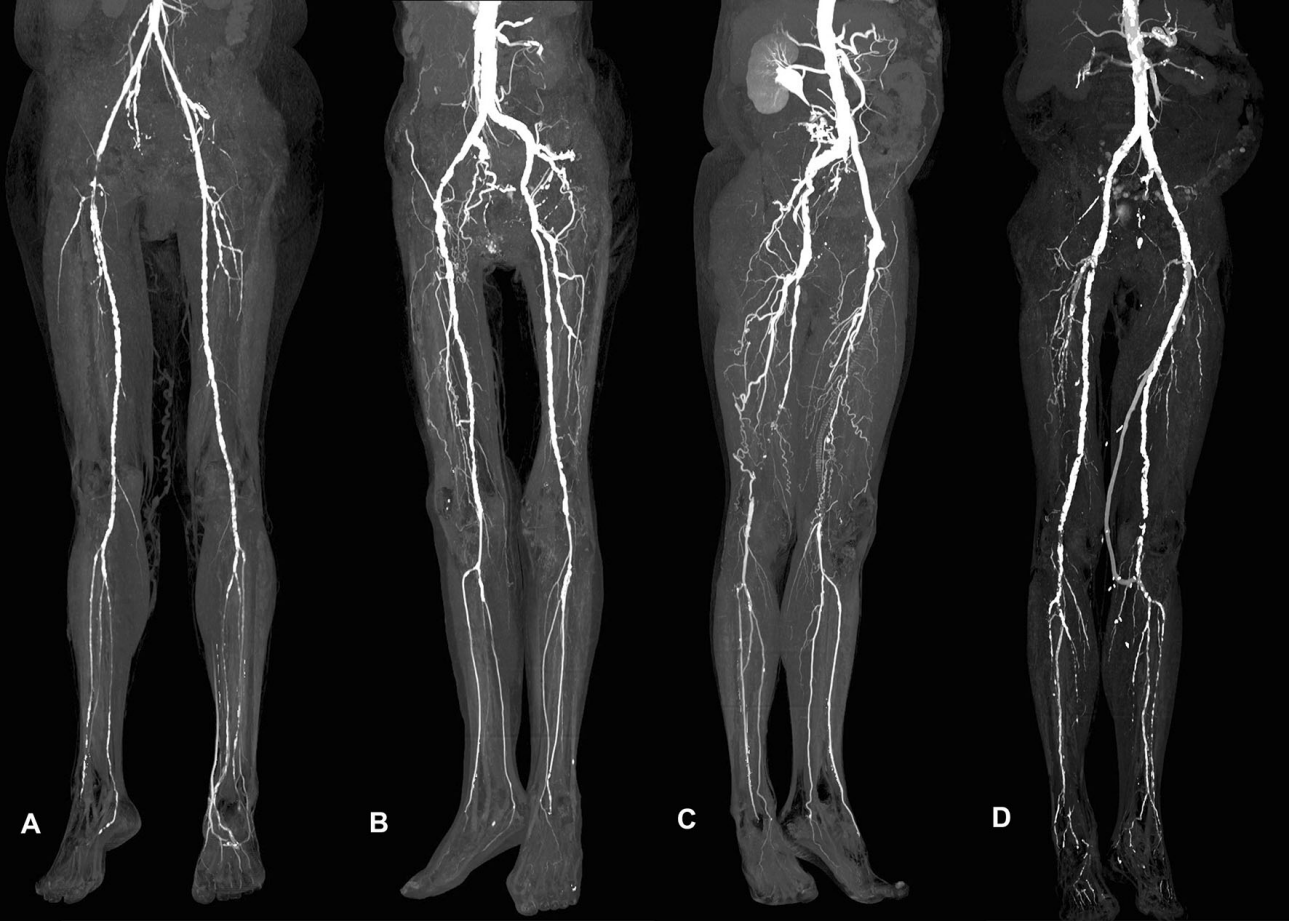
Maximum intensity projections (MIP) of the lower limb CTA after bone subtraction. **A** Right SFA occlusion in 46-year-old female causing Rutherford I symptoms. **B** Significant stenosis of right CIA in 57-year-old female patient with metastatic cervical carcinoma, referred for CTA with suspected thromb-embolic source in the peripheral arteries. Multiple vascular caliber changes of CIA and SFA are visible bilaterally. **C** 64-year-old male patient with left CFA aneurysm (6.8 × 4.6 cm), occluded left femoro-popliteal bypass and bilateral SFA occlusion including proximal PA (level P1). **D** Rutherford III complaints in 66-year-old male patient caused by stenosis of left femoro-popliteal bypass at the level of distal anastomosis and complete right SFA occlusion. *CFA* common femoral artery, *CIA* common iliac artery, *CTA* computed tomographic angiography, *SFA* superficial femoral artery, *EIA* external iliac artery, *PA* popliteal artery

To the best of our knowledge, this is the first study reporting on feasibility of diagnostic CTA of the lower extremities in patients with PAD using only a 45 ml CM volume protocol (including the 15 ml test bolus), with the lowest attributed total iodine load (TIL;13.5 gI). Similar studies with a 70 kV setting for CTA of the lower extremities were performed on a first generation DSCT, with 80 ml of CM (300 mgI/ml) and a TIL of 24 gI [10]. Baxa et al. performed lower extremity CTA with similar CM volumes (10 ml test and 40 ml main bolus); however, the TIL in their study was 48% higher compared to our study, due to the higher concentration of CM (400 mgI/ml). The CM administration is often feared by the clinicians, especially in patients at risk of contrast-induced nephropathy (CIN) development. However, referral for an unenhanced CT study may compromise the additive diagnostic value of imaging. As Nijssen et al. [22] recently showed that prophylactic intra-venous hydration before and after CM administration in patients with estimated glomerular filtration *(*eGFR) of 30–59 mL/min/1.73 m^2^ is non-superior to no prophylaxis, the focus in CIN prevention now lies in optimizing CM administration. The use of minimal volumes of pre-warmed nonionic low-osmolar CM is considered to reduce the risk of CIN, even in patients with pre-existing renal insufficiency [23], and allows safe and effective CM use in high-risk patients [22]. However, the reduction in contrast volume should not compromise adequate intravascular enhancement. In this study, the administration of an ultra-low CM volume resulted in diagnostic image quality even in patients with severe PAD (Rutherford II and III) or acute vascular occlusion, allowing for accurate diagnosis and selection of appropriate interventional strategy (Fig. 4).

**Fig. 5:**
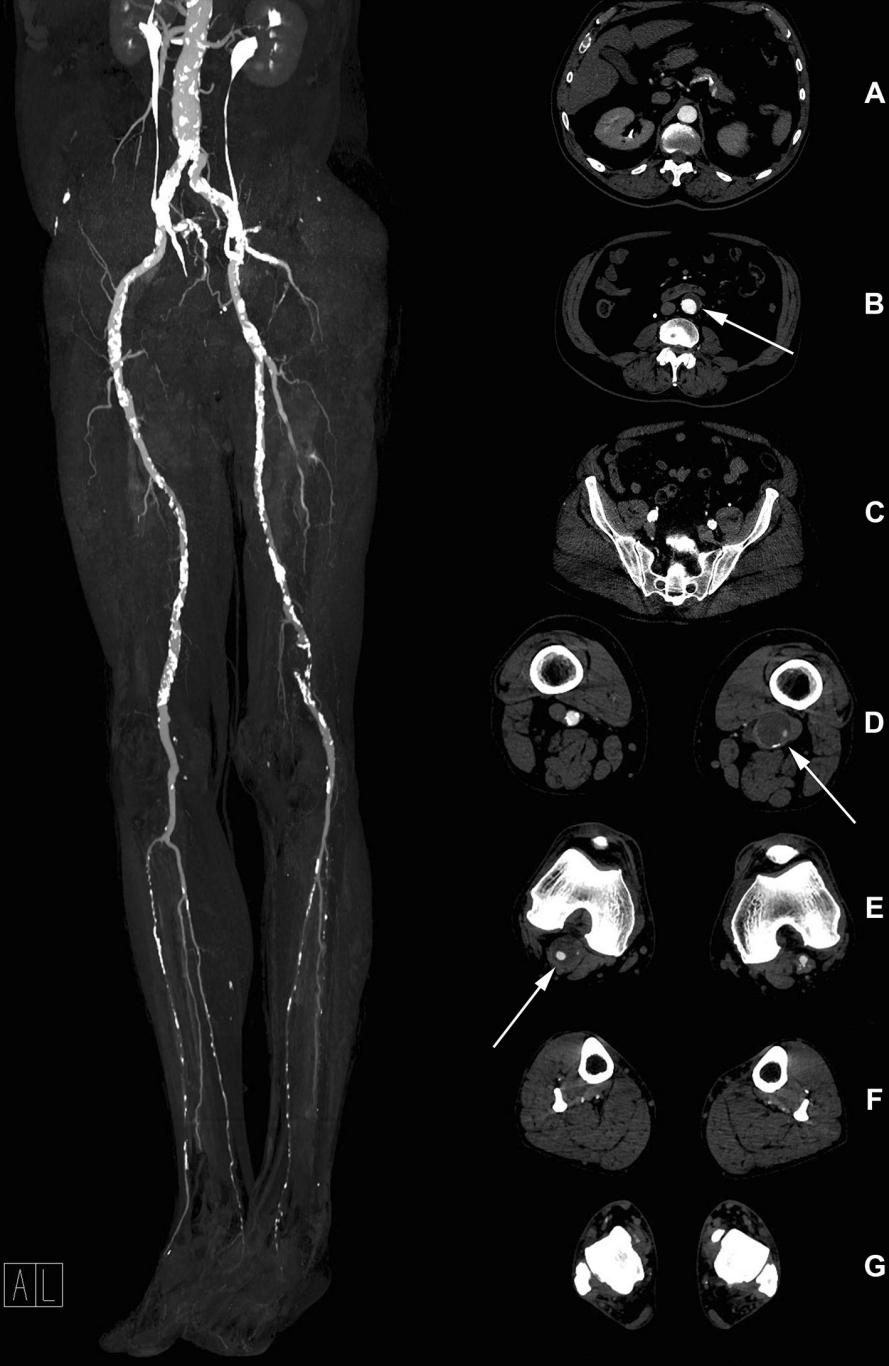
Peripheral vascular run-off in 75-year-old male patient with infra-renal aortic and bilateral popliteal aneurysm (left occluded), significant stenosis of right proximal EIA and SFA bilaterally, and compromised lower leg vasculature. Good flow in the left CIA stent. White arrows indicate aneurysmatic changes of infra-renal abdominal aorta (**B**) and PAs (**D** and **E**). CM attenuation is visible in the crural (**F**) and pedal arteries (**G**). *CIA* common iliac artery, *CM* contrast media, *EIA* external iliac artery, *SFA* superficial femoral artery, *EIA* external iliac artery, *PAs* popliteal arteries)

**Fig. 6:**
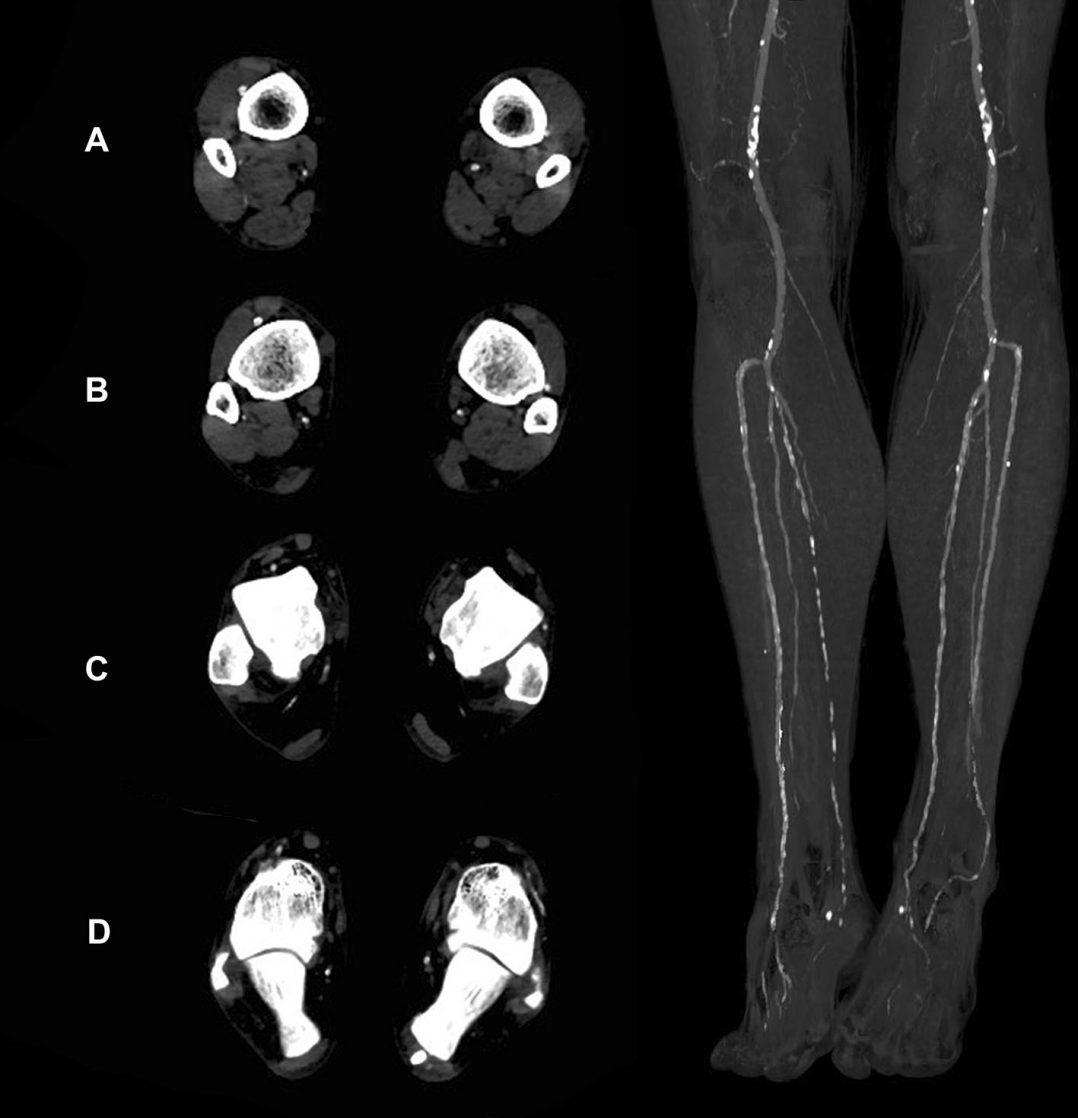
Peripheral vascular run-off in 77-years-old male patient presenting with multiple level stenoses (bilateral IIA, left CIA and EIA, right SFA). Transversal images show CM attenuation in the crural (**A** and **B**) and pedal arteries (**C** and **D**). *CIA* common iliac artery, *CM* contrast media, *EIA* external iliac artery, *IIA* internal iliac artery, *SFA* superficial femoral artery)

To achieve diagnostic image quality, intravascular attenuation has to be sufficient in all evaluated segments. With a mean scan range of 125 cm, the short injection time of the main bolus, lasting only 6 s, imposes a risk of outrunning the short duration of the attenuation peak. Presence of an aneurysm, bypass, vascular stenosis or occlusion may significantly influence the bolus transit time and increase the difference between extremities, or extensively delay the propagation of contrast enhancement in the distal segments (Fig. 5) [24, 25]. The difference in the mean attenuation values between the left and right DPA vasculature therefore most likely reflects the pathophysiological phenomenon rather than a technical difficulty with the protocol used in our study. Moreover, in all above-located segments the mean difference between the left and right-sided attenuation was not significant, despite of the unequal distribution of vascular pathologies or their severity. Furthermore, the presence of a vascular lesion, independent of severity, did not influence the achieved level of intravascular attenuation. Although the ANOVA showed a statistically significant difference in attenuation between TASC II lesion groups in right aorto-iliacal segment, the mean difference of 5 HU between non-diseased and most severely affected (type D) segments should not be considered clinically relevant. The contrast media distribution delay due to the vascular pathophysiology can also explain the dynamic decrease in the subjective image quality from the PA (excellent) to the crural arteries (good), which were mostly superior to DPA segments (graded as good or diagnostic) (Fig. 6). However, to the best of our knowledge, the image quality of DPA was assessed in studies performed with higher CM volumes (100 ml of 400 mgI/ml [26]) or when a dedicated lower leg acquisition was performed [27], while none of the recently published studies evaluated feasibility of low CM volume CTA of lower extremities assessed the image quality in pedal arteries [10, 28–30].

Fleischmann et al. [25] recommend to prolong the injection duration with a corresponding increase in the scan delay to allow an adequate filling distal to the occluded segment. Overall high attenuation values suggest the possibility to reduce CM concentration by mixing the main bolus with saline in order to increase injection volume to prolong the injection time, while the TIL stays the same. The optimum CM volume, concentration, iodine delivery rate (IDR) and injection time for satisfactory attenuation in DPA need to be clarified in a further study. However, the cases with high complete vascular occlusion at the level of CIA or SFA demonstrated that the double-level test bolus technique is able to overcome such severe occlusion, while delivering diagnostic attenuation in all PA and 10/11 ATA segments.

Patients with PAD may undergo repeated imaging on various occasions during the course of their disease. Thus, a great attention was directed toward not only the reduction in CM volume, but also the acquired radiation dose. Qi et al. in 2014 used the combination of a 70kVp and high-pitch (2.2) acquisition to lower radiation dose down to 0.3 ± 0.1 mSv. However, this very low ED only quantifies the acquired radiation dose between the pelvic crest and proximal third of the thighs [10]. Other studies, using low kV scan protocols, also calculated the ED from the region between abdominal aorta and iliac arteries resulting in 5.5 ± 0.9 mSv at 80 kV and 1.94 ± 0.21 mSv at 70 kV [29, 30]. Baxa et al. [28] calculated an ED of 3.9 mSv from the total scanning distance at 100 kV. In our study, the whole-body ED (1.1 ± 0.5 mSv) was assessed using dedicated software based on a Cristy phantoms library, taking into account patients diameter, age and weight [16, 31]. Increasing the pitch value may help to further decrease the ED [7]; however, artifacts due to helical interpolation at the image reconstruction process may occur. These artifacts are most likely to appear at the level of popliteal trifurcation, where relatively small diameter vessels branch and therefore deviate rapidly off the longitudinal (z-axis) direction [12]. On the other hand, low pitch (in this study ranged between 0.35 and 0.70), in combination with iterative reconstruction, can partly compensate for an increased image noise resulting from the low-tube-voltage setting. Even further decrease in ED, beyond the levels achieved in our study, may not be necessary, since Nguyen et al. [32] recently reported no evidence of DNA damage associated with activation of genes involved in regulating cell repair and programmed cell death in patients undergoing coronary CTA when radiation doses of ≤ 7.5 mSv were used. Thus, the low dose approach may be especially beneficial in cases of premature PAD [33] or in patients with a combined diagnosis of PAD and diabetes mellitus, as they are more likely to be repeatedly referred for CTA examinations earlier in life [34].

There are some limitations in this study. The major limitation of our study is the lack of a gold standard to which the results of our study have been compared; however, previous literature has already reported an excellent sensitivity and specificity of lower tube voltage CTA in comparison with DSA in assessment of PAD [29]. Second a relatively small patient population was evaluated in this feasibility study and therefore comparably low count of TASC II lesion types was present in some segments. Third, the laboratory criteria for renal function evaluation, in terms of eGFR and serum creatinine (sCr) levels before and 3–5 days after contrast administration in CTA, were not available in most of the patients. Therefore, no conclusions can be drawn from this study in regard to the CIN development. Fourth, the assessments of the peak attenuation times in Syngo DynEvaTM and the calculation of individual scan time and scan delay require additional attention and time compared to the uniform acquisition independent of patient’s characteristics. Fifth, the bone removal software often subtracts the small diameter vessels enhancement in the distal lower leg [35]. Therefore, the segmented maximum intensity projection (MIP) images may not be reliable at this level. Sixth, in most cases this protocol is not suitable for patients with large lower extremity metal implants, because of beam hardening and scatter artifacts [11]. Reliable evaluation is then difficult to impossible in certain segments. Lastly, comparison with a CTA protocol previously used in our institution was not performed, as double protocol would significantly increase the TIL and radiation dose in referred patients.

## Conclusion

A low-tube-voltage scan protocol allows for a significant reduction of the injected CM volume and the acquired radiation dose, while maintaining sufficient objective and subjective image quality for the evaluation of PAD, regardless of the lesion severity.

## Abbreviations

ATA: Anterior tibial artery
ANOVA: Analysis of variance
CI: Confidence interval
CIA: Common iliac artery
CIN: Contrast-induced nephropathy
CM: Contrast media
CNR: Contrast-to-noise ratio
CT: Computed tomography
CTA: Computed tomographic angiography
DLP: Dose-length product
DPA: Dorsalis pedis artery
DSA: Digital subtraction angiography
DSCT: Dual-source computed tomography
ED: Effective dose
eGFR: Estimated glomerular filtration rate
gI/l: Grams of iodine per liter
HU: Hounsfield Unit
ICRP: The International Commission on Radiological Protection
MIP: Maximum intensity projection
PA: Popliteal artery
PAD: Peripheral artery disease
PTA: Percutaneous transluminal angioplasty
ROI: Region of interest
sCr: Serum creatinine
SD: Standard deviation
SFA: Superficial femoral artery
SNR: Signal-to-noise ratio
TASC II: Trans-Atlantic Inter-Society Consensus Document II
TIL: Total iodine load
